# A two-years real-word study with fingolimod: early predictors of efficacy and an association between EBNA-1 IgG titers and multiple sclerosis progression

**DOI:** 10.3389/fimmu.2024.1384411

**Published:** 2024-06-07

**Authors:** Maria Inmaculada Dominguez-Mozo, Victoria Galán, Lluís Ramió-Torrentà, Ana Quiroga, E. Quintana, Luisa María Villar, Lucienne Costa-Frossard, José Ignacio Fernández-Velasco, Noelia Villarrubia, María Angel Garcia-Martinez, Rafael Arroyo, Roberto Alvarez-Lafuente

**Affiliations:** ^1^ Grupo de Investigación de Factores Ambientales en Enfermedades Degenerativas, Instituto de Investigación Sanitaria del Hospital Clínico San Carlos (IdISSC), Red de Enfermedades Inflamatorias (REI), Madrid, Spain; ^2^ Servicio de Neurología, Hospital Universitario de Toledo, Toledo, Spain; ^3^ Neuroimmunology and Multiple Sclerosis Unit, Girona Biomedical Research Institute (IDIBGI), Doctor Josep Trueta University Hospital and Santa Caterina Hospital, Department of Medical Sciences, University of Girona, Red de Enfermedades Inflamatorias (REI), Girona, Spain; ^4^ Neuroimmunology and Multiple Sclerosis Unit (UNIEM), Girona Biomedical Research Institute (IDIBGI), Red de Enfermedades Inflamatorias (REI), Girona, Spain; ^5^ Girona Neuroimmunology and Multiple Sclerosis Unit (UNIEM), Girona Biomedical Research Institute (IDIBGI), Department of Medical Sciences, University of Girona, Girona, Spain; ^6^ Servicio de Inmunología, Hospital Universitario Ramón y Cajal, Red de Enfermedades Inflamatorias (REI), Madrid, Spain; ^7^ Servicio de Neurología, Hospital Universitario Ramón y Cajal, Red de Enfermedades Inflamatorias (REI), Madrid, Spain; ^8^ Departamento de Neurología, Hospital Universitario Quironsalud Madrid, Red Española de Esclerosis Múltiple (REEM), Madrid, Spain

**Keywords:** multiple sclerosis, fingolimod, Epstein-Barr virus, human herpesvirus 6, human leukocyte antigen

## Abstract

**Background:**

Although fingolimod, a sphingosine 1-phosphate receptor agonist, has shown to be an effective treatment reducing relapse rate and also slowing down the disability progression in relapsing-remitting multiple sclerosis (RRMS) patients, it is important to quickly identify those suboptimal responders.

**Objective:**

The main objective was to assess different clinical, radiological, genetic and environmental factors as possible early predictors of response in MS patients treated with fingolimod for 24 months. The secondary objective was to analyze the possible contribution of the environmental factors analyzed to the progression and activity of the disease along the 2-years of follow-up.

**Methods:**

A retrospective study with 151 patients diagnosed with MS, under fingolimod treatment for 24 months, with serum samples at initiation and six months later, and with clinical and radiological data at initiation and 24 months later, were included in the study. Clinical and radiological variables were collected to establish NEDA-3 (no evidence of disease activity: patients without relapses, disability progression and new T2 lesions or Gd+ lesions) and EDA (evidence of disease activity: patients with relapses and/or progression and/or new T2 lesions or gadolinium-positive [Gd+] lesions) conditions. Human leukocyte antigen II (HLA-II), EBNA-1 IgG and VCA IgG from Epstein-Barr virus (EBV) and antibody titers against Human herpesvirus 6A/B (HHV-6A/B) were also analyzed.

**Results:**

A total of 151 MS patients fulfilled the inclusion criteria: 27.8% was NEDA-3 (37.5% among those previously treated with high efficacy therapies >24 months). The following early predictors were statistically significantly associated with NEDA-3 condition: sex (male; p=0.002), age at baseline (older; p=0.009), relapses 2-years before fingolimod initiation ≤1 (p=0.010), and absence of Gd+ lesions at baseline (p=0.006). Regarding the possible contribution of the environmental factors included in the study to the activity or the progression of the disease, we only found that EBNA-1 IgG titers decreased in 20.0% of PIRA (progression independent from relapse activity) patients vs. 73.3% of RAW (relapse-associated worsening) patients (p=0.006; O.R. = 11.0).

**Conclusion:**

MS patients that are male, older, and with a low clinical and radiological activity at fingolimod initiation have a greater probability to reach NEDA-3 condition after two years with this therapy. An intriguing association of EBV with the progression of the disease has also been described, but it should be further study in a larger cohort to confirm these results.

## 1 Introduction

Fingolimod is a sphingosine 1-phosphate (S1P) receptor agonist that significantly reduces disease activity in relapsing-remitting multiple sclerosis (RRMS) patients (1). This oral immune‐modulatory treatment embodies and degrades the sphingosine‐1‐phosphate (S1P) receptor on leukocytes, inhibiting the egress of lymphocytes from lymph nodes. Therefore, this therapy is able to reduce the migration of potential inflammatory cells to the central nervous system; as a consequence, patients under fingolimod treatment present peripheral blood lymphopenia due to the redistribution of leukocytes (2). Although the U.S. Food and Drug Administration (FDA) approved this treatment for RRMS patients, its use in Europe was restricted to highly active RRMS patients or as escalation after failure to first‐line disease modifying therapies (DMT) (3). In the FREEDOMS I study, the 70.4% of MS patients treated with the 0.5mg dose, was free of relapses after 24 months of follow-up (1); these results were very similar to those of the FREEDOMS II performed later: 71.5% (4). Regarding real-world studies, results are similar to those obtained in the clinical trials or even better when the percentage of relapse-free patients is analyzed, after two years of fingolimod treatment: 81.9% of 286 Turkish MS patients (5), 77.4% of 78 Italian MS patients (6), 74.6% of 167 Spanish MS patients (7). Therefore, although fingolimod has shown to be an effective treatment reducing relapse rate and also slowing down the disability progression, it is important to quickly identify those suboptimal responders.

With this aim, we performed a retrospective study analyzing different clinical, radiological, genetic and environmental factors as possible early predictors of response in MS patients treated with fingolimod along 24 months. Some clinical factors have been previously related to fingolimod response as early predictors (8, 9). Regarding genetic factors, they have been searched before in relation to the response to the different DMTs in MS. Pharmacogenomic studies have shown that the human leukocyte antigen (HLA) could help to better identify appropriated candidates to each treatment (10, 11), although in other occasions not (12, 13). In fact, a recent paper published in pediatric and adolescent MS patients treated with fingolimod identified different haplotypes that showed a trend towards a more favorable clinical course (14). Finally, viruses have been proposed as possible biomarkers of response to different DMTs (15, 16). Viruses like Human herpesvirus 6 (HHV-6) or Epstein-Barr virus (EBV) have been proposed to be actively involved in processes like the activity (17) or the progression of the disease (18, 19), and therefore, modifications in their antibody levels or in their viral load along the treatment could be identified in relation with these processes. The main objective of this study was to assess all of them, in the same cohort of MS patients treated with fingolimod, in relation to the clinical and radiological response comparing NEDA-3 (no evidence of disease activity: patients without relapses, disability progression and new T2 lesions or Gd+ lesions) and EDA (evidence of disease activity: patients with relapses and/or progression and/or new T2 lesions or Gd+ lesions) patients. The secondary objective was to analyze the possible contribution of the environmental factors analyzed to the progression and activity of the disease along the 2-years of follow-up. Furthermore, in the last years, different S1P receptor agonists have been approved for the treatment of MS patients (20–22). Thus, the results obtained with fingolimod could be of interest for them, since they largely share the same mechanism of action.

## 2 Methods

### 2.1 Design

This is a retrospective study with the following inclusion criteria: 1) MS patients diagnosed by Poser, 2010 or 2017 McDonald criteria (23–25); 2) under fingolimod treatment for 24 months; 3) with serum samples collected within a month before starting fingolimod treatment and six months after the first dose; 4) with the following clinical and radiological data at initiation and 24 months later: Expanded disability status scale (EDSS) score, number of relapses, T2 lesions and gadolinium-positive (Gd+) lesions at initiation, and one and two years later.

### 2.2 Patients

Patients were recruited from the following hospitals: Hospital Clínico San Carlos (Madrid), Hospital Universitario Doctor Josep Trueta (Gerona) and Hospital Universitario Ramón y Cajal (Madrid). The clinical and radiological data were collected by neurologists and radiologists belonging to the Multiple Sclerosis Units of these hospitals.

### 2.3 Ethics statement

This study was approved by the local Ethic Committee of the Hospital Clínico San Carlos (Comité Ético de Investigación Clínica del Hospital Clínico San Carlos). All the patients recruited received and signed a written informed consent. All experiments were performed in accordance with relevant guidelines and regulations.

### 2.4 Response criteria

According to the pre-treatment EDSS score, we defined the progression as the following increases after 24 months of fingolimod treatment: 1) EDSS=0: ≥1.5 points; 2) EDSS ≥1 and ≤5: ≥1 point; 3) EDSS ≥5.5: ≥0.5 points. We also analyzed two different variables related to the progression: RAW (relapse-associated worsening: patients with progression and with at least one relapse during the two years of follow-up under fingolimod treatment) and PIRA (progression independent from relapse activity: patients with progression and without relapses during the two years of follow-up under fingolimod treatment). Although they are non-mutually exclusive drivers for long-term disability, we considered PIRA and RAW as two competing outcomes (26). Relapses were defined as a worsening of neurological impairment or an appearance of a new symptom or abnormality attributable to MS; they lasted at least 24 hours and they were preceded by stability period of at least 1 month. Regarding magnetic resonance imaging (MRI), it was performed one month prior fingolimod treatment onset and 1 and 2 years after starting this therapy in 1.5T scanners; a previous published protocol was followed (27). The sequences collected for this study were; axial fluid-attenuated inversion recovery (FLAIR) T2, axial proton density T2-weighted imaging, axial T2-weighted imaging, and T1-weighted imaging with gadolinium (Gd) enhancement. With previous definitions, these were the response criteria: clinical response (absence of disability progression and relapses), NEDA-3 (no evidence of disease activity: patients without relapses, disability progression and new T2 lesions or Gd+ lesions), and EDA (evidence of disease activity: patients with relapses and/or progression and/or new T2 lesions or Gd+ lesions).

### 2.5 Researched variables

The following variables were analyzed: 1) Clinical: sex, age at recruitment, age at disease onset, disease duration, number of relapses two years before the recruitment, EDSS score at treatment initiation and previous treatments. Therapies were classified as: moderate efficacy treatments (MET: beta-interferon, glatiramer acetate, dimethyl fumarate and teriflunomide) and high efficacy treatments (HET: natalizumab, mitoxantrone, azathioprine) 2) Radiological: T2 and Gd+ lesions at recruitment and after one and two years of fingolimod treatment. 3) Genetic: HLA-II. 4) Viral: antibody responses to EBV (EBNA-1 IgG and VCA IgG) and HHV-6 IgG and IgM antibody titers at fingolimod initiation; based on previous published results of our group we also analyzed the change in the antibody titers between the baseline and the six month sample (15, 17).

### 2.6 DNA extraction

DNA spin column technique of QIAamp DNA Blood Mini Kit (QIAGEN. Hilden. Germany) was used to isolate total DNA from 0.2 ml of blood, following the manufacturer’s instructions.

### 2.7 HLA genotyping

The genotyping of the alleles belonging to HLA class II (DR, DQA and DQB) was carried out using the rSSO-PCR technique (sequence-specific oligonucleotide reverse PCR). LABType™ One Lambda kits (Thermo Fisher Scientific, Waltham, MA, USA) were used following the manufacturer’s instructions. The detection was carried out on a FLEXMAP 3D™ device (Luminex Corporation, Austin, TX, USA), whose detection is based on Luminex™ technology.

### 2.8 ELISA

Commercial tests for the detection of EBNA-1 and VCA IgG (Trinity Biotech, USA) and HHV-6A/B IgG and IgM (Vidia, Ltd., Czech Republic) were used in an automated ELISA processing system (DS2, Dynex Technologies, USA), following the manufacturer’s instructions. Results were expressed in artificial units (AU): index value * 10 (index value = sample absorbance/cut-off value) (15, 17). Each sample was analyzed in duplicate for each test. Doubtful samples (between 9 and 11 AU) were tested again; they were considered negative samples if they remained under 11 AU at the new analysis.

### 2.9 Statistical analysis

To test differences in categorical variables we used the chi-square or two-tailed Fisher’s exact test. To analyze differences in continuous variables the Kruskall-Wallis test or the Wilcoxon rank-sum test were used. Association between clinical, radiological, genetic and environmental factors as possible early predictors and clinical outcomes (activity and/or progression of the disease), alone or in combination (NEDA-3/EDA) was studied with a non-parametric test (U Mann-Whitney). Bonferroni correction was performed when multiple comparisons were made. P-values <0.05 were considered as statistically significant. All statistical analysis were performed using Statistical Package for Social Sciences, version 15.0 (IBM SPSS, Inc, Chicago, IL, USA).

## 3 Results

### 3.1 Patients recruited for the study and demographic characteristics of the population study

A total of 151 MS patients fulfilled all the inclusion criteria of the study. Their characteristics are shown in **Table 1**.

**Table 1:** Demographical characteristics of the patients included in the study at the onset of fingolimod treatment.

Sex	N
Males	46
Females	105
**Age [years, med (P25-P75)]**	38.6 (33.0–45.0)
**Age at disease onset [years, med (P25-P75)]**	26.0 (22.0–31.0)
**Disease duration at fingolimod onset [months, med (P25-P75)]**	125.0 (75.0–190.9)
**EDSS [med (P25-P75)]**	3.0 (2.0–4.0)
**Relapses 2 years before [med (P25-P75)]**	1.0 (0.0–2.0)
**Patients with at least one relapse 2 years before [n/N(%)]**	102/151 (67.5%)
**Treatment naïve (N)**	9
Last treatment before fingolimod onset	N
Glatiramer acetate	27
Interferon beta	38
Mitoxantrone	3
Azathioprine	1
Natalizumab	71
Dimethyl fumarate	1
Teriflunomide	1
Duration of the last treatment [months, med (P25-P75)]	29.0 (16.5–47.5)

(med, median; P25, 25th percentile; P75, 75th percentile; EDSS, Expanded Disability Status Scale)

### 3.2 Clinical and radiological response after two years of fingolimod treatment

The 34.4% (52/151) of MS patients suffered relapses after two years of fingolimod treatment vs. 67.5% (102/151) two years prior to fingolimod onset (49% of reduction). The 19.9% (30/151) of MS patients experienced progression according to the progression criteria above mentioned: 50% of them were PIRA (15/30) and 50% were RAW (15/30). Regarding the MRI studies, the 38.4% (58/151) of patients had new T2 lesions after 24-months of fingolimod treatment; the 21.2% (32/151) had Gd+ lesions at 12-month and/or 24-month MRI. According to our response criteria, the 55% (83/151) could be considered as clinical responders and the 27.8% (42/151) as NEDA-3 after two years of fingolimod treatment.

### 3.3 Early clinical and radiological variables

**Figure 1:**
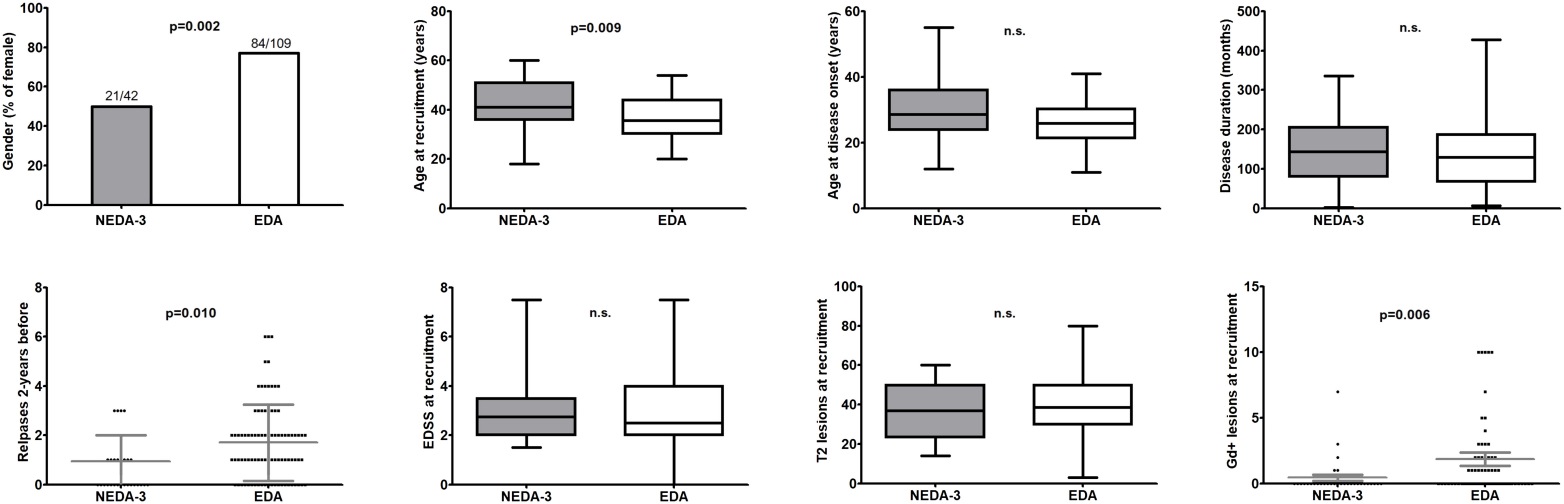
Comparison of clinical and radiological variables between NEDA-3 and EDA patients at recruitment (p values were calculated from Chi-square test/Fisher’s exact test for categorical variables and with the Kruskall-Wallis test for the continuous variables). Lines inside the graphs shows the mean value. (GraphPad Prism 5.0) n.s., not significant.

When we compared MS patients with NEDA-3 vs. those with EDA, the following early predictors were statistical significantly associated with NEDA-3 condition (**Figure 1**): sex (male; p=0.002), age at baseline (older; p=0.009), relapses 2-years before fingolimod initiation ≤1 (p=0.010), and absence of Gd+ lesions at baseline (p=0.006). Thus, the 71.4% of those MS patients with these four early predictors were NEDA-3 vs. the 6.7% among those MS patients without any of them. Since most of the MS patients had been previously treated (**Table 1**), we analyzed their possible relation with the number of relapses two years before and with the number of Gd+ lesions at fingolimod initiation. We found statistically significant differences when we compared MS patients treated with MET and HET for more than 24 months (**Table 2**): a higher proportion of MS patients previously treated with HET had ≤1 relapses 2-years before (p=0.003; O.R. = 5.3) and no Gd+ lesions (p=<0.0001; O.R. = 12.3) at fingolimod initiation. However, when we analyzed the effect of MET and HET treatments prior to fingolimod initiation on NEDA-3 condition, we did not find any statistically significant difference (**Table 3**).

**Table 2:** Effect of previous treatments on the number of relapses two-years before fingolimod initiation and on the number of Gd+ lesions at baseline samples.

	Treated 3–12 months				Treated 13–24 months				Treated >24 months			
	MET	HET	p*	O.R.**	MET	HET	p*	O.R.**	MET	HET	p*	O.R.**
**patients with ≤1 relapse** **2-years before fingolimod initiation**	6/16 (37.5%)	2/8 (25.0%)	n.s.		5/16 (31.3%)	11/17 (64.7%)	0.06	4.0	18/32 (56.3%)	41/47 (87.2%)	0.003	5.3
**Mean of relapses 2-years before fingolimod initiation**	2.3	2.4	n.s.		1.9	1.6	n.s.		1.3	0.6	0.002	
**patients without Gd+ lesions at baseline sample**	11/16 (68.8%)	5/8 (62.5%)	n.s.		13/16 (81.3%)	13/17 (76.5%)	n.s.		13/32 (40.6%)	42/47 (89.4%)	<0.0001	12.3
**Mean of Gd+ lesions at baseline sample**	0.5	1.0	n.s.		0.9	0.6	n.s.		0.5	0.0	0.002	

*p values were calculated from Chi-square test/Fisher’s exact test. **Odds Ratios. MET, moderate efficacy treatments (beta-interferon, glatiramer acetate, dimethyl fumarate and teriflunomide). HET, high efficacy treatments (natalizumab, mitoxantrone, azathioprine). n.s., not significant.

**Table 3:** Effect of previous treatments on NEDA-3 condition.

Naïve MS patients	Treated MS patients	0–12 months	13–24 months	25–48 months	>48 months	p* (O.R.**) MET vs. HET
NEDA-3		NEDA-3	NEDA-3	NEDA-3	NEDA-3	
3/9 (33.3%)	**MET**	3/19 (15.8%)	5/16 (31.3%)	3/17 (17.6%)	3/15 (20.0%)	0.100 (1.9)
	**HET**	1/11 (9.1%)	6/17 (35.3%)	11/31 (35.5%)	7/16 (43.8%)	
	p* (O.R.**)	0.607 (1.9)	0.806 (1.2)	0.202 (2.6)	0.166 (3.1)	
	p* (O.R.**)	0.843 (1.1)		0.069 (2.7)		

*p values were calculated from Chi-square test/Fisher’s exact test. **Odds Ratios. MET, moderate efficacy treatments (beta-interferon, glatiramer acetate, dimethyl fumarate and teriflunomide). HET, high efficacy treatments (natalizumab, mitoxantrone, azathioprine).

### 3.4 HLA-II as early biomarker of response

After Bonferroni correction, we only found statistically significant associations for DR4 carriers: most of them were clinical responders (p=0.008; O.R. = 3.4) and none of them progressed after two-years of follow-up (p=0.006; O.R. = 15.9). However, the statistical significance was not reached when analyzing the NEDA-3 condition (p=0.09; O.R. = 2.6).

### 3.5 Viral serologies and the response to fingolimod treatment

**Figure 2:**
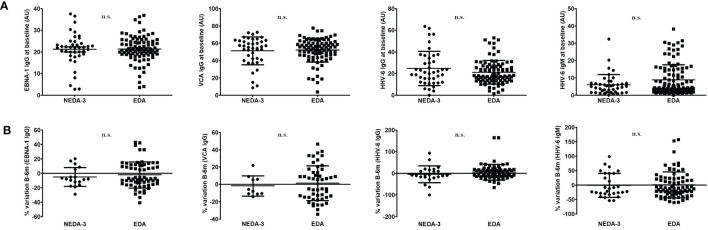
**(A)**. EBNA-1 IgG, VCA IgG or HHV-6 IgG and IgM titers at baseline in NEDA-3 and EDA patients. **(B)** Variation in the antibody titers between NEDA-3 and EDA patients (we did not consider variations ≤4.8%, our inter-assay coefficient of variation). Significations with the two-tailed t-test are shown (n.s., not significant). Lines inside the graphs shows the mean value plus the standard deviation. (GraphPad Prism 5.0).

**Figure 3:**
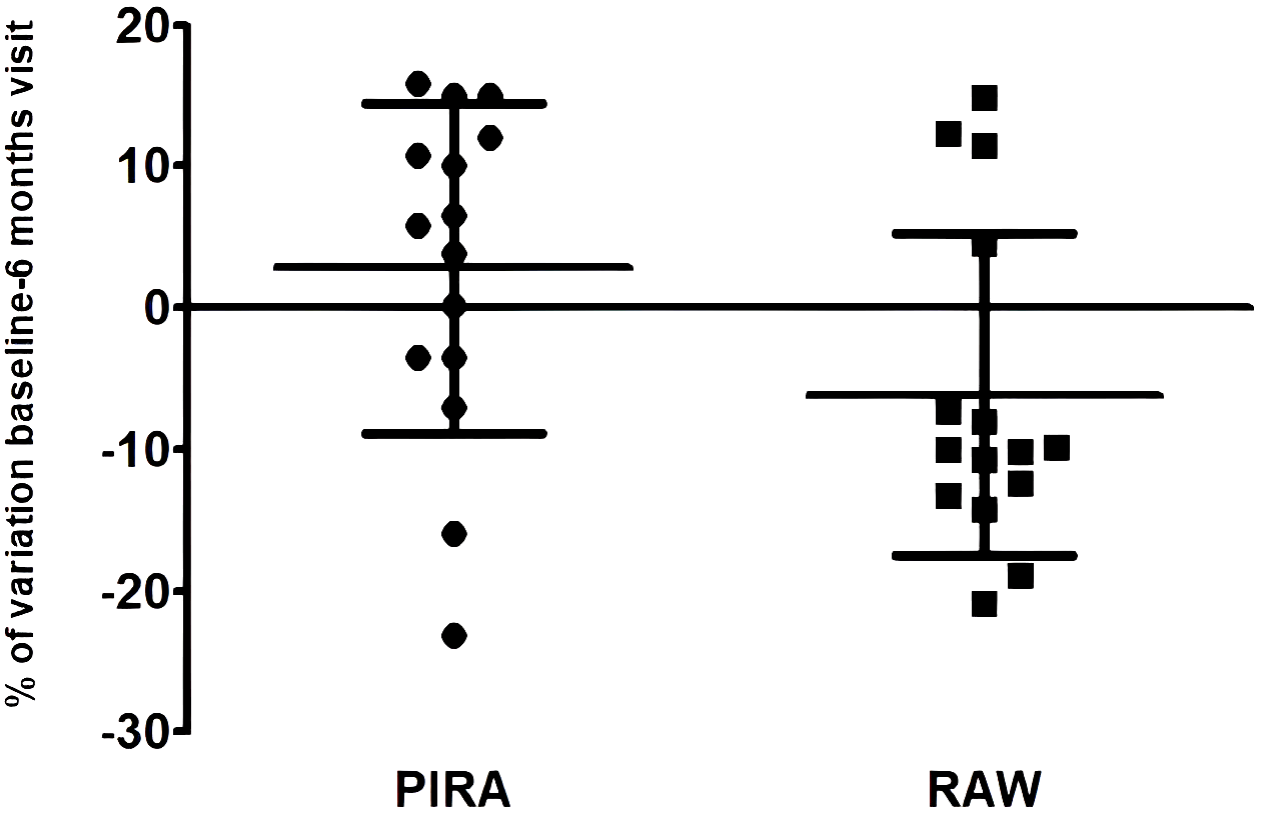
EBNA-1 IgG variation between the baseline sample and the six month sample in PIRA and RAW patients. Mean values plus standard deviation (GraphPad Prism 5.0).

We did not find statistically significant associations for EBNA-1 IgG, VCA IgG or HHV-6 IgG and IgM titers at baseline with NEDA-3 condition (see **Figure 2A**). The change in the antibody titers between the baseline and the six month sample was not associated either (**Figure 2B**); we did not consider variations ≤4.8% (our inter-assay coefficient of variation). Finally we also analyzed the possible association of the baseline antibody titers and the antibody titers variation between the baseline sample and the six month sample with the progression (yes vs. no, and PIRA vs. RAW) and with the activity of the disease (with relapses vs. without relapses, and with Gd+ lesions vs. without Gd+ lesions), after 2-years of follow-up with fingolimod treatment. Again, we did not consider variations ≤4.8% (our inter-assay coefficient of variation). After Bonferroni correction for multiple comparisons, we only found one statistically significant association when we compared PIRA and RAW patients: we found that EBNA-1 IgG titers decreased in 3/15 (20.0%) PIRA patients after two-years of fingolimod therapy vs. 11/15 (73.3%) RAW patients (p=0.006; O.R. = 11.0) (**Figure 3**).

## 4 Discussion

Identifying early predictors of response in MS is essential for improving patient outcomes, optimizing treatment strategies, and advancing our understanding of the disease. It holds the potential to transform MS care by enabling personalized, timely, and effective interventions, ultimately benefiting both patients and the healthcare system as a whole. With this purpose, we analyzed different clinical, radiological, genetic and serological variables to search for early predictors of NEDA-3 condition after two-years of treatment with fingolimod.

Patients recruited for this multicenter study showed a lower proportion of NEDA-3 condition compared to most of the studies performed in real-world: 56.6% (28), 44% (29), 48.3% (8), 44% (30), but similar to other real-world studies, 22% (31), and *post-hoc* analyses of randomized clinical trials, 29.5% (32), and 31.0% (33). The variability in NEDA-3 proportion could be explained by the heterogeneity of MS patients included in the different real-world studies. In our cohort, only 9/151 (6.0%) were treatment naïve, and previous studies published a higher proportion of NEDA-3 condition in treatment naïve patients treated with fingolimod (28). Regarding the other patients of our study, 67/151 (44.3%) were treated with MET (median: 25.0 months; range: 1–192 months) and 75/151 (49.7%) were treated with HET (median: 32.0 months; range: 1–92 months). As it has been previously described (28), previous DMTs influence the NEDA-3 proportion of MS patients treated with fingolimod. In our study, although a clear effect of previous treatments was described on the number of relapses and Gd+ lesions prior fingolimod initiation (**Table 2**), no statistically significant differences were found in relation to the NEDA-3 condition (**Table 3**). However, trends were found when we compared MET vs. HET, and also when we compared MS patients treated with MET and HET for more than two years, showing that not only prior treatments but also their duration may influence the subsequent clinical response to fingolimod.

In our study, the following early predictors were statistically significant associated with NEDA-3 condition: sex (male), age at baseline (older), relapses 2-years before fingolimod initiation ≤1, and absence of Gd+ lesions at baseline. The last three have been yet previously reported as early predictors of fingolimod treatment (8), together with lower EDSS score at baseline (9). However, previous studies have not found significant differences in treatment response between males and females taking fingolimod. In our cohort, 21/46 (45.7%) males were NEDA-3 vs. 21/105 (20.0%) females. No differences in previous treatments were found between males and females, and we did not find any difference for the other three variables between them either. Further studies will be needed to find a possible explanation.

Regarding the genetic variables analyzed, we found that DR4 carriers were better clinical responders and none of them progressed after two-years of follow-up. There is only one previous study analyzing different genetic data to find predictors of response to fingolimod (20), but none analyzing HLA apart from one searching for HLA alleles that differentially regulate John Cunningham (JC) virus antibody serostatus in MS patients treated with fingolimod (34). The term DR4 refers to the HLA-DRB1*04, that is associated with an increased risk of developing MS, mainly in some Mediterranean populations (35). Regarding MS, HLA-DRB1*04 alleles have been associated with primary progressive MS (PP-MS) (36); however, other studies could only either suggest a non-significant trend to a positive association of HLA-DRB1*04 alleles with PP-MS (37), or no effect at all (38). The possible association of HLA-DRB1*04 with PP-MS and the results of our study showing absence of progression in DR4 carriers treated with fingolimod, suggest further studies to solve this intriguing association.

Finally, we described an interesting association between EBNA-1 IgG titers and the progression of the disease. The 73.3% of RAW patients experienced a decrease of those antiviral titers between baseline visit and six-month visit after two years of fingolimod treatment vs. only 20.0% of PIRA patients. EBV is currently considered one of the main risk factors in MS (39). A recent study showed that an immune response against EBV could turn against the host, triggering disease progression (40). Different studies have supported the role of EBV in the progression of the disease in the last years (41). Here we first describe a different behavior of EBV depending on the different progression of the disease. We could speculate that EBV would be associated to those mechanisms involved in the progression of the disease that are independent of relapse activity, also referred to as smoldering MS (42), rather than in the relapse-associated worsening. The re-analysis of previous clinical trials or observational studies from the perspective here described of a different behavior of EBV in PIRA and RAW patients could be very valuable.

However, one limitation of the study could be its retrospective design. Thus, we should be aware about possible selection bias of the patients recruited for the study, and therefore, the level of evidence could be inferior compared with prospective studies. Other limitation of the study, related to one of the significant results obtained, could be the small size of the cohorts resulting from dividing those MS patients who progressed after two years of fingolimod treatment, between PIRA and RAW. It would also have been desirable to have a measurement of the EBV antibody titers in serum samples collected at 24 months to confirm the titer variations obtained at the 6 month visit.

In conclusion, here we describe four early predictors that could help to identify optimal responders to fingolimod treatment. Thus, according to the current study, MS patients that are male, older, and with a low clinical and radiological activity at fingolimod initiation have a greater probability to reach NEDA-3 condition after two years with this therapy. An intriguing association of EBV with the progression of the disease has also been described, but it should be further study in a larger cohort to confirm these results.

## Data availability statement

The original contributions presented in the study are included in the article/**Supplementary Material**. Further inquiries can be directed to the corresponding author.

## Ethics statement

The studies involving humans were approved by Comité Ético de Investigación Clínica del Hospital Clínico San Carlos. The studies were conducted in accordance with the local legislation and institutional requirements. The participants provided their written informed consent to participate in this study.

## Author contributions

MD-M: Formal analysis, Investigation, Methodology, Writing – original draft, Writing – review & editing, Validation. VG: Writing – review & editing, Resources. LR-T: Resources, Writing – review & editing. AQ: Resources, Writing – review & editing. EQ: Resources, Writing – review & editing. LV: Resources, Writing – review & editing. LC-F: Resources, Writing – review & editing. JF: Resources, Writing – review & editing. NV: Resources, Writing – review & editing. AM: Investigation, Methodology, Writing – review & editing. RA: Resources, Writing – review & editing. RA-L: Conceptualization, Data curation, Formal analysis, Funding acquisition, Investigation, Methodology, Supervision, Writing – original draft, Writing – review & editing.
